# Polarization Processes in Nafion Composite Membranes Doped with Rare-Earth Metals

**DOI:** 10.3390/ma16186172

**Published:** 2023-09-12

**Authors:** Rene Castro, Elena Karulina, Nikolay Lapatin

**Affiliations:** Institute of Physics, Herzen State Pedagogical University of Russia, 48 Moika Emb., 191186 St. Petersburg, Russia; recastro@mail.ru (R.C.); karulina@mail.ru (E.K.)

**Keywords:** perfluorosulfonic membrane, modification, sorption, rare-earth elements (Eu^3+^, Tb^3+^), electrophysical properties, polarization processes, dielectric spectroscopy, relaxator parameters, polymer matrix, charge transfer

## Abstract

Dielectric spectroscopy (frequency range f = 10^0^…10^7^ Hz and temperatures T = 293…403 K (accuracy 0.5 K), measuring voltage applied to the sample was 1.0 V) was used to study composite materials based on perfluorosulfonic membranes with inclusions of rare-earth elements, in particular, europium (III) and terbium (III) chlorides. The dispersion of the permittivity and the presence of maxima, corresponding to losses, were revealed, which indicates that relaxation processes of various natures were present. The membrane layers under investigation are characterized by relaxation parameters that correspond to a symmetrical distribution of relaxers over relaxation times. The spectrum of relaxers changed when terbium and europium metal impurities were introduced into the polymer matrix. The investigation of these polymer systems demonstrated a power-law dependence of the specific conductivity on frequency. A decrease in the exponent with increasing temperature indicates the existence of a traditional hopping mechanism for charge transfer. The observed changes in the dielectric permittivity and specific conductivity are due to a change in the nature of polarization processes because of the strong interaction of metal (terbium and europium) ions with the polymer matrix of Nafion.

## 1. Introduction

Polymeric membranes and the processes occurring within them have been extensively studied for several decades. However, the production of synthetic polymeric membranes has become merchantable only in recent years. The industrial development of their production and usage have become a real opportunity to be seized. Membranes are used in various processes of separation and concentration of substances in liquid and gas phases. The comparative simplicity and efficiency of membrane processes ensure their widespread usage [1,2,3,4,5,6,7,8].

Perfluorosulphonic membranes (PFSMs) represent a distinct class of ionomers. According to the Gierke model, the membrane contains a system of spherical clusters connected by branched channels, the inner surface of which contains sulfonic groups. The commercial name Nafion is given to PFS membranes, first obtained and patented by DuPont (Nafion ion exchange membranes are fundamentally different from polymer analogs, for example, polytetrafluoroethylene, that do not contain ionogenic groups). In the second half of the 1960s, the Nafion membrane was discovered by Walther Grot and patented by du Pont de Nemours (Wilmington, DE, USA). At present, a number of Nafion analogs have been developed, such as “Flemion” (Asahi Glass Co., Tokyo, Japan), “Aciplex-S” (Asachi Glass Co., Tokyo, Japan), “Dowmembrane”, and “Neosepta” (Tokuyama Soda Inc., Tokyo, Japan), which are products of Asahi Chemical (Asahi Glass Co., Tokyo, Japan) (Aciplex), Asahi Glass (Tokyo, Japan) (Flemion), FuMA-Tech (Bietigheim-Bissingen, Germany) (Fumapem), Dow Chemical (Midland, MI, USA), and others, with only minor differences in structure and number of units. For example, Dow has proposed a membrane with a shorter side chain than Nafion [9].

Perfluorosulphonic membranes are widely used as nanoreactors for various reactions, in particular, alkylation, esterification, dehydration, acylation and isomerization, etc. [10,11].

The hydrogen sorption capacity of [11] paracyclophane functionalized with Li is investigated using density functional theory. Li functionalized [11] paracyclophane physisorbs 8 H_2_, achieving a maximum hydrogen weight percentage of up to 13.42% [12].

The proton exchange membrane (PEM) made of perfluorosulphonic acid is extensively used in direct methanol fuel cells (DMFCs) as a proton carrier, methanol barrier, and separator for the anode and the cathode. Nafion has a significant disadvantage due to the high permeability of the methanol fuel from the anode to the cathode. PEM is also commonly used in flow batteries. For flow batteries, there are also crossover issues of active metallic species through the membrane; hence, self-discharge takes place easily during operation. The Nafion membranes’ functional properties can be improved by introducing inorganic fillers, carbon nanomaterials, ionic liquids, polymers, etc. [13,14,15,16,17,18,19,20,21,22,23,24,25,26,27,28,29,30].

The investigation of the membrane structure of PFS membranes like Nafion, quantitative and qualitative description of the size and structure of the inner surface, as well as the position of the sulfonic acid group often leads to contradictory results. The Nafion membrane is usually considered as a three-component system. It contains three parts: a tetrafluoroethylene-like principal chain, a fluorinated side chain that terminates in a sulfonic acid group, and absorbed water. The authors of [21] applied the method of small-angle neutron scattering. At large scales (>30 nm), structural heterogeneities were observed in the backbone and side chain domains, but not in the water domains. The medium scale (5–30 nm) demonstrates a biconvex structure of the crystalline and amorphous phases with an average separation distance of 11 nm, due to the semi-crystalline template effect of the backbone chain. On a small scale (<5 nm), another biconvex structure exists in the amorphous phase with an average separation distance of about 4 nm, indicating a well-connected water network responsible for the high conductivity of the membrane. A cross-sectional analysis of the PSF for the two components revealed the location of each component, indicating that the backbone chain domains tend to phase separate from either side chain domains or water domains. However, the side chain and water domains are closely linked through sulfonic acid groups.

It is noteworthy that membranes possess a unique capability that enables the self-organization of the structure and substances encapsulated in the pore space of the membranes during their sorption by the polymer membrane surface. A set of inert perfluorinated carbon chains forms the basis of the membrane. The functional groups and water associated with them are combined into small islands, which are called clusters in the literature [6].

The spatial structure organization of PFS membranes determines the main properties that make them suitable for a wide range of practical applications. The data indicate that the size of the pore space in PFSMs is a few nanometers. This fact makes them related to nanostructured objects [21,22]. Thus, materials constructed based on PFS membranes exhibit the most prevalent characteristics of nanosystems, and, above all, the so-called size effect (the significant dependence of a sample’s property on dimension parameters).

It is interesting to note that among the literature data, there are no attempts at the direct (heterogeneous) potentiometric titration of PFS membranes with alkali. The known content of sulfonic acid group in PFS membranes [–SO_3_H] is 0.84 ÷ 0.95 mmol/g [23] (obtained by the back titration method). However, there is evidence to suggest that the manifestation of the membrane as a strong acid is possible.

In recent years, many attempts have been made to investigate the electrophysical properties of membranes. Special attention is paid to the water effect on the change in dielectric parameters, so in [24], the authors studied the dielectric properties of Nafion membranes in the high-frequency area. A high dependence of the dielectric constant of the membrane on the water content was found. In [25], the spectra of thermally stimulated depolarization (TSD) were studied in the temperature range of 77–300 K; the presence of three dispersion regions I-III and the dependence of their position on the water content were noted. Region I is similar to the *γ*-relaxation mechanism observed in mechanical relaxation studies, possessing an activation energy between 0.55 and 0.68 eV. Region II is similar to the *β*-relaxation mechanism observed in studies of mechanical relaxation on hydrated membranes. Region III can be explained by a phase or structural change in the temperature range under study. The results of experiments with different electrode configurations were presented, revealing various aspects of the relaxation mechanism’s dynamic behavior and distinguishing between the contribution of dipolar and interfacial polarization of dispersion II.

Low-frequency dielectric relaxation has been studied in a wide frequency range [26,27]. The authors of [26] used the four-probe method, which allows researchers to exclude interfacial polarization on the electrode with the polymer. The volume conductivity at direct current and the permittivity of Nafion membranes were determined to be equal to *λ*~0.03 *S* cm^−1^ and *ε′*~106 (*T* = 40 °C and *RH* = 100%).

It is known that the proton in the Nafion membrane is rather easily replaced by other cations (Li^+^, Na^+^, K^+^, Mg^2+^, etc.). It is impossible to improve the transport characteristics of systems based on a modified Nafion membrane with metal cation conductivity, expand their operating temperature range without establishing the electrotransport mechanisms, or evaluate the role of various factors (the nature of the cation, the nature and amount of the dopant, etc.) in ensuring fast ion transport. However, the information available in the literature is extremely scarce, even for Li-Nafion. There is even less information in the literature for other cations, which does not allow for the creation of a generalized model of ion transport and understanding its mechanisms.

In the specialized literature, a special place is occupied by studies of composite materials based on polymeric membranes doped with rare-earth elements [31,32].

A manifestation of unusual spectral–luminescent properties was found. It should be expected that rare-earth metals should also affect the dielectric properties of membranes and change the conductivity of systems. In this regard, the purpose of this work was to reveal the features of polarization processes and their relationship with the structure in Nafion films doped with rare-earth metals by the dielectric spectroscopy method.

## 2. Structural Studies

The analogue of the Nafion membrane, MF-4SK (OJSC “Plastpolymer”, Saint-Petersburg, Russia), 220.0 ± 0.5 µm thick and 1–2 cm^2^ in area, is the object of study.

**Figure 1:**
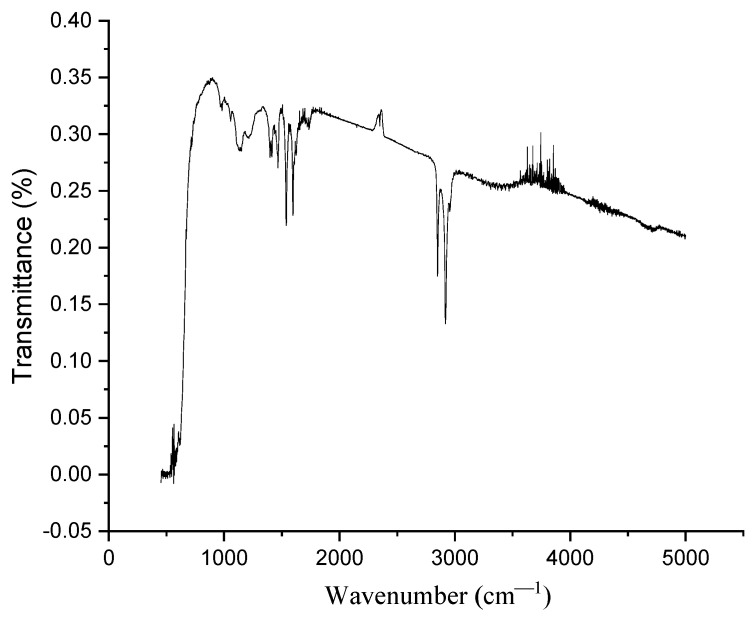
ATR-spectrum of Nafion membranes.

The optical properties and structure of the polymer samples were studied by Fourier-IR spectroscopy on an FSM-1202 Fourier spectrometer with an ATR attachment, operating in the range of 400–5000 cm^–1^. Figure 1 demonstrates the IR-spectrum of the studied membranes. The maxima of the transmission bands of fluorocarbon chains are observed as follows: 625, 717, 970, 982, 1200, 1375, 1414, 1456, and 2924 (overtone) cm^−1^, belonging to the membrane. Peaks 1057 and 1144 cm^−1^ correspond to symmetric stretching vibrations, while 1312 and 1445 cm^−1^ correspond to asymmetric stretching vibrations that belong to the sulfonic acid group.

**Figure 2:**
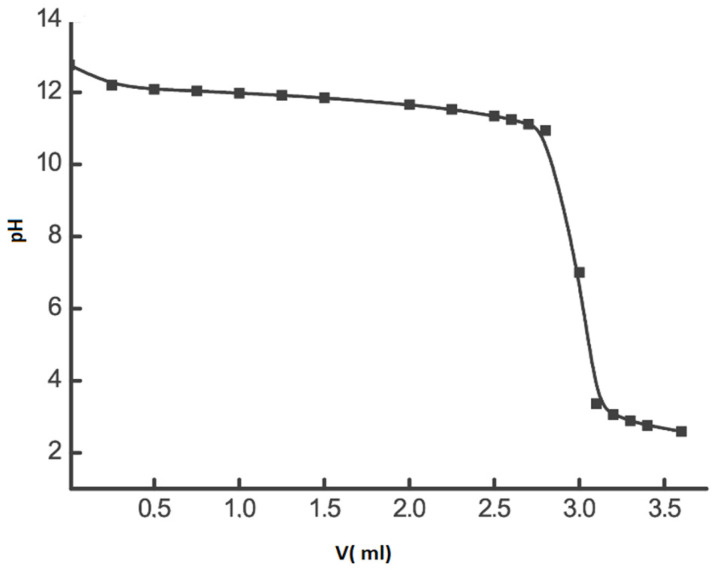
Typical view of the residual alkali titration curve after contact of NaOH solution (20 mL; 0.0192 mol/L) with an MF-4SK membrane (0.1 g); concentration of HCl titration solution is 0.1 mol/L.

The content of sulfonic acid groups via the loss of alkali in the solution because of its contact with the membrane was determined by the method of back titration (Figure 2). The value obtained from the results was [–SO_3_H] = 0.84 ± 0.05 mmol/g.

The content of sulfonic acid group in the membrane was also determined by using pH control. The membranes were pre-dried in air at 90 °C to constant mass and placed in a glass with distilled water. NaOH solution was added dropwise while stirring. The process of replacing the protons of the sulfonic acid group with sodium cations was observed by value changes recorded by the pH-meter “Anion 4100”. An abrupt change in the titration curve indicates the complete neutralization of the acid centers in the membrane. The concentration of alkali at the equivalence point (pH~6.7) corresponds to the number of the sulfonic acid group in the membrane. The value established by the results of 5 determinations was [–SO_3_H] = 0.84 ± 0.04 mmol/g.

**Figure 3:**
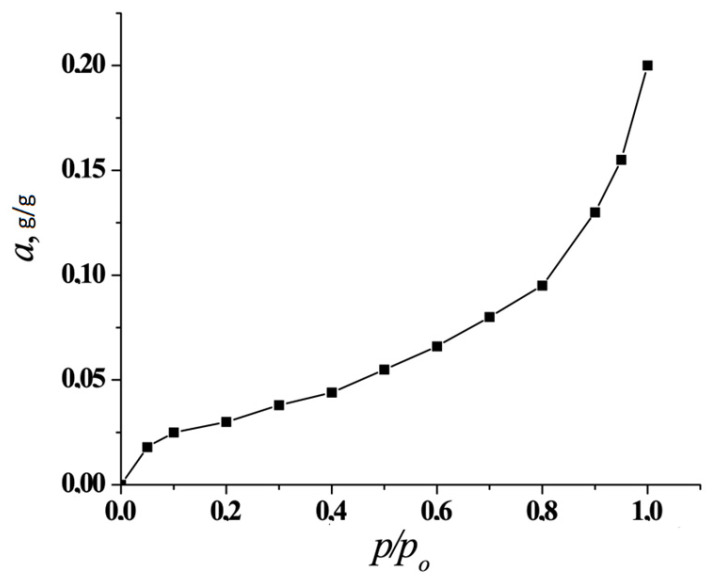
Isotherm of water adsorption by the MF-4SK membrane; *a*—adsorption value, *p*/*p_o_*—relative vapor pressure.

Adsorption measurements were carried out in order to obtain information about the location of functional groups in the membrane. Figure 3 shows the adsorption isotherm. The weakly expressed S-shaped form of the isotherm and the numerical values of water adsorption make it possible to estimate the value of the specific surface area (SSA) of the studied Nafion membranes.

It is not possible to determine the true value of the specific surface area of the membrane, since it is impossible to ensure complete dehydration of the membranes (90 °C) while maintaining the original porous structure. Nevertheless, an approximate estimation of the magnitude of SSA seems to be justified. The value of adsorption *a_m_* ≈ 0.04 g/g at *p*/*p_o_* = 0.25 was taken as the conditional capacity of a water monolayer (Figure 3). In this case, taking into account the landing area of the molecule *ω* = 10 Ǻ^2^ leads to a very high result.

*SSA* = *a_m_*∙*ω*∙*N_A_* ≈ 130 (m^2^/g), (1)

According to the S-shaped form of the adsorption isotherm, the process of water condensation in the membrane pores begins in the area of *p*/*p_o_* = 0.7 − 0.8. The final point of the isotherm *p*/*p_o_* = 0.99 (Figure 3) corresponds to the capillary/volumetric filling of pores. According to the results of measurements, the mass gain relative to the dry state (at 90 °C) characterizes the available sorption volume of the membrane, which is *V_n_* = 0.20 ± 0.03 cm^3^/g.

By comparing the values of SSA ≈ 130 m^2^/g and [–SO_3_H] = 0.84 mmol/g, an idea of the topography of the arrangement of functional groups can be received. The average value of the area per one sulfonic acid group is ~25 Ǻ^2^ in this case, indicating a significant distribution density of sulfonic acid groups on the surface of the PFSM.

The distribution topography and the high activity of the membrane sorption centers determine the maximum filling of its surface according to the following scheme:

3(–SO_3_H) + La^3+^ → (–SO_3_–)_3_ La + 3H^+^. (2)

Taking into account that the membrane sulfonic acid groups are fully used in ion exchange substitution processes, the assessment of the areas occupied by Tb^3+^ and Eu^3+^ cations gives ~75 Ǻ^2^, which corresponds to an average distance between them of ~9 Ǻ. The result obtained is similar to the above estimate of the distribution of –SO_3_H groups and serves as an indication of the unusually high sorption activity of the MF-4SK membrane.

**Figure 4:**
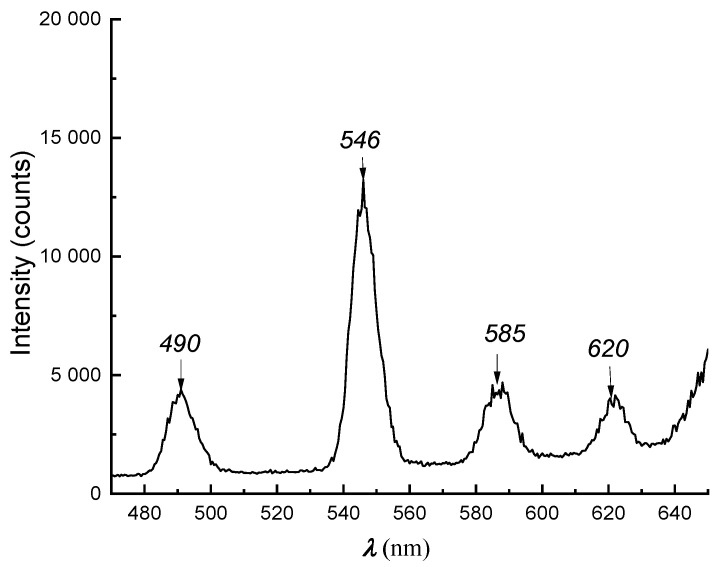
Luminescence spectrum of Tb^3+^ in MF-4SK membrane (*λ_ex_* = 219 nm).

In order to verify the presence of terbium (III) and europium (III) in the investigated composite membranes, the luminescence spectra were measured on an SF-6000 spectrofluorimeter (Shimadzu, Kyoto, Japan) at an excitation length of 219 nm. Figure 4 demonstrates the luminescence spectrum of the Nafion membrane modified with Tb^3+^.

The luminescence spectrum of the membrane modified with Tb^3+^ demonstrates four out of seven bands, which can be associated with radiative transitions from the excited ^5^*D*_4_ to the ground states of terbium ^7^*F*_j_ (j = 6, 5, 4, 3). The good resolution of the spectrum indicates that the coordination environment of the cation has a high symmetry. The band with a maximum at 546 nm, which has the maximum intensity, corresponds to the ^5^*D*_4_ → ^7^*F*_5_ transition, which is called supersensitive. The maximum at 219 nm of the only excitation band of Tb^3+^ coincides with its position in the absorption spectrum of the cation in solution [23,33,34].

The membrane modification with lanthanide salts (TbCl_3_ and EuCl_3_) from water solutions proceeds according to the following scheme:

3(–SO_3_H) + LaCl_3_ + nH_2_O = (–SO_3_–)_3_La (H_2_O)_n_ + 3HCl, (3)

The stability of membranes modified with rare-earth metal ions is confirmed by the preservation of the luminescence and absorption spectra shapes.

The sulfonic acid groups at the terminal ends of the side chains of PFS membranes are extremely acidic due to the influence of electron-withdrawing –CF_2_ groups, which contributes to the easy electrostatic binding of cations. When the membrane is treated with acid, it becomes saturated with protons and passes into the H-form. The protons’ ability to move in the water, which fills the pore space of the membrane, results in a high proton conductivity.

Thus, the structural analysis of the investigated Nafion composite films allows us to draw conclusions about the similarity between the Russian analog of the MF-4SK membrane and Nafion, as well as the high activity of membranes in the processes of ion exchange sorption.

## 3. Dielectrical Studies

The samples under study were the layers of the Nafion membrane—MF-4SK. The layer was 220.0 µm thick and 1–2 cm^2^ in area. The membrane was preliminarily cleaned from impurities sorbed from the air by boiling it for several hours in a HNO_3_ solution (65% by mass). The studied samples then were washed in aqua distillata from excess NO_3_^−^, and dried to a constant weight at a temperature of 90 °C.

Dielectric spectra (temperature–frequency dependences of dielectric parameters) were measured using a Concept-81 spectrometer (core shared research facilities for Dielectric Spectroscopy of Herzen State Pedagogical University). The measurements were carried out in the frequency range of *f* = 10^0^…10^7^ Hz and temperatures of *T* = 293…403 K (accuracy 0.5 K). The measuring voltage applied to the sample was 1.0 V.

The values of the imaginary and real components of the complex impedance of the cell with the measured sample were taken as experimental data. The complex impedance is given by

(4) $$Z^{*}(\omega )=R+\frac{1}{i\omega C}=Z^{\prime }+iZ^{\prime \prime }=\frac{U_{0}}{I^{*}(\omega )},$$

The complex permittivity and complex conductivity spectra are calculated from the impedance spectra using the following formulas:

(5) $$\varepsilon^{*}=\varepsilon^{\prime }-i\varepsilon^{\prime \prime }=\frac{-i}{\omega Z^{*}(\omega )}\frac{1}{C_{0}},$$

where $C_{0}=\frac{\varepsilon_{0}S}{d}$ is the capacity of the empty cell and $C=\frac{\varepsilon \varepsilon_{0}S}{d}$ is the capacity of the cell with the sample.

In turn, the following relation expresses the complex conductivity in terms of the complex impedance:

(6) $$\sigma^{*}=\sigma^{\prime }-i\sigma^{\prime \prime }=\frac{-i}{\omega Z^{*}(\omega )}\frac{S}{d},$$

The experiment and calculation relative error of the electrical parameters of the investigated layers did not exceed 5%.

### 3.1. Dielectric Relaxation

**Figure 5:**
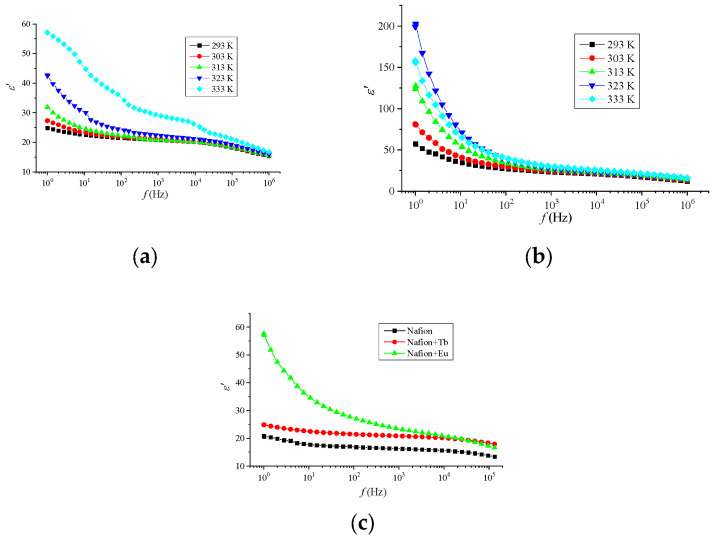
Frequency dependence of the permittivity of Nafion + Tb (**a**), Nafion + Eu (**b**) system layers at different temperatures, Nafion (**c**) (293 K).

Figure 5 demonstrates the frequency dependence of the permittivity ε′ for the layers of three systems at different temperatures. It is clear that ε′ decreases with increasing frequency, ranging from 20 to 200 at low frequencies.

The decrease in *ε′* with frequency is caused by the decrease in the contribution of the dipole-orientational polarization, since it begins to take more time than other types of polarization (electronic, ionic, space charge polarization). The dipoles are not able to turn fast enough, and their rotation lags behind the change in the electric field. As the frequency continues to increase, the dipole will be unable to follow the field and orientational polarization will cease. Thus, *ε′* decreases, approaching a constant value at high frequencies, due to the contribution of only the electronic polarization and the space charge polarization [35].

**Figure 6:**
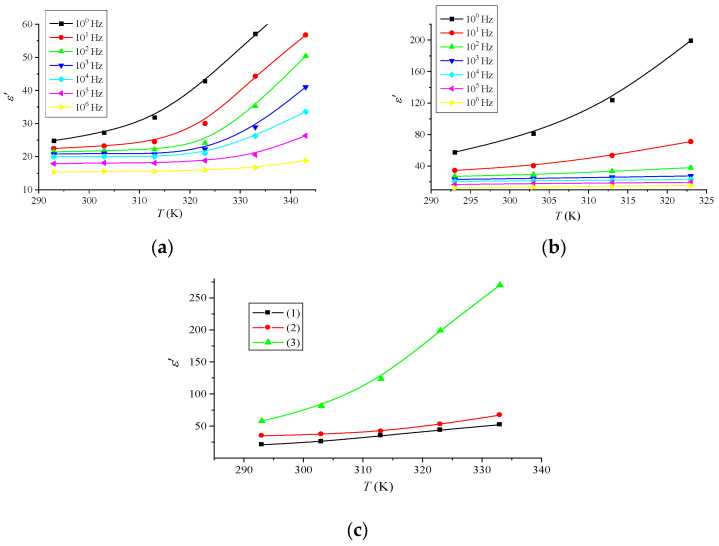
Temperature dependence of the permittivity of the Nafion + Tb (**a**); Nafion + Eu (**b**) system for different frequencies of the applied alternating field; Nafion systems doped with rare-earth metals at a frequency f = 100 Hz (**c**): (1)—Nafion, (2)—Nafion + Tb, (3)—Nafion + Eu.

Figure 6 shows the temperature dependence of the permittivity *ε′*. It can be seen from the figure that *ε′* exhibits a similar growth pattern to the temperature. The increase in *ε′* with temperature can be attributed to the fact that dipoles in polar materials cannot self-orientate at low temperatures. As the temperature increases, the dipoles’ orientation becomes easier, the orientational polarization increases, and consequently, *ε*′ increases.

The observed increase in the dielectric constant of the Nafion system doped with terbium or europium can be attributed to the strong polarizing effect of metal ions on water molecules in the polymer matrix and, as a consequence, an increase in the number of protons in the system. For example, for a terbium impurity, such an interaction can be carried out according to the following scheme:

[Tb(H_2_O)n]^3+^ ↔ [Tb(H_2_O)n-1 OH]^2+^ + H^+^, (7)

In many materials, relaxation processes are associated with the existence of not only one relaxation time, but a whole set of relaxation times. In this case, it can be argued that there exists a distribution of relaxation times and, consequently, activation energies. This distribution can be attributed to the manifestation of relaxation processes of different nature or the distribution of dipole concentrations within the structure. In the case of ion hopping processes, it is assumed that the potential energy changes after each hop, and it takes some time for potential energy to return to its lowest level. Taking into account the contribution of numerous mobile defects, a set of relaxation times is obtained.

**Figure 7:**
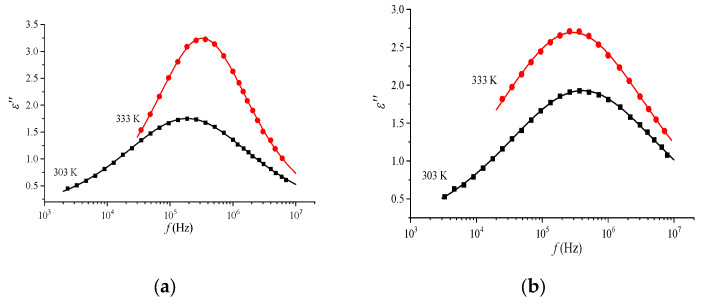
Frequency dependence of loss factor for Nafion (**a**) and Nafion + Tb (**b**) and layers at two temperatures.

The investigation of the frequency dependence of the loss factor *ε″* for samples of three systems at different temperatures (Figure 7) revealed the existence of maxima, corresponding to losses, which shift in frequency with increasing temperature. The presence of maxima on the *ε″*(*f*) curves in the studied frequency range indicates the existence of relaxation processes that cause relaxation losses in the systems [36].

The shape of the dielectric loss factor curve, *ε″* = *f*(*ν*), or the dielectric loss tangent, tg*δ* = *f*(*ν*), can be used to estimate the distribution of relaxation times [37]. In this paper, the values of the relaxation parameters were obtained by approximating the experimental curves using the framework of the Havriliak–Negami approximation [35]:

(8) $$\varepsilon^{*}(\omega )=\varepsilon_{\infty }+\frac{\Delta \varepsilon }{{\left[1+{\left(i\omega \tau \right)}^{\alpha_{HN}}\right]}^{\beta_{HN}}},$$

where *ε*_∞_ is the high-frequency limit of the real component of the permittivity, Δ*ε* is the dielectric increment (the difference between the low-frequency and high-frequency limits), *ω* = 2π*ν*, *α*HN and *β*HN are the shape parameters describing, respectively, the symmetric (*β* = 1 − Cole–Cole distribution) and asymmetric (α = 1 − Cole–Davidson distribution) extension of the relaxation function. The obtained values of relaxation parameters (Table 1) confirm the assumption about the existence of a distribution of relaxers over relaxation times. Pure (undoped) Nafion membranes are characterized by values of relaxation parameters that correspond to a symmetrical distribution of relaxers over relaxation times (beta is equal to one). Beta takes on values different from one with the introduction of europium and terbium for most temperatures. It is a sign of an asymmetrical distribution of relaxers over relaxation times. Nafion membranes are characterized by values of relaxation parameters that correspond to a symmetrical distribution of relaxers over relaxation times. When a metal impurity is introduced into the polymer matrix, the spectrum of relaxers undergoes a change, which is manifested in the predominantly asymmetric nature of their distribution.

It can be assumed that –CF_2_, –CH, and –CH_2_ groups, as well as hydrophilic functional polar sulfonic acid groups –SO_3_, detected by infrared scanning of the membranes, can act as relaxers [38].

**Figure 8:**
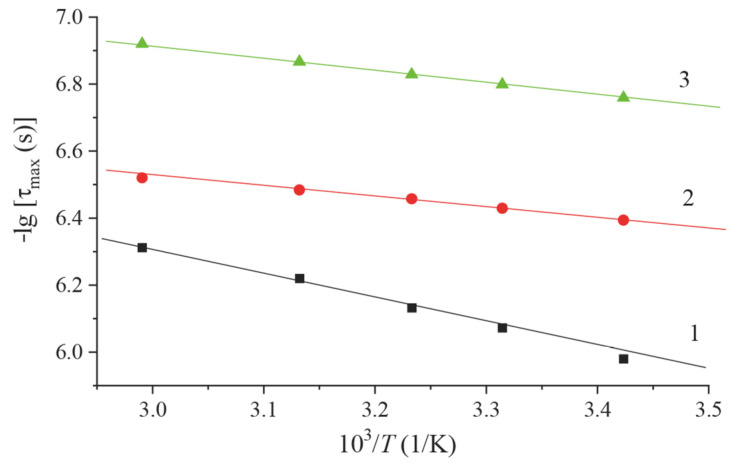
Temperature dependence of the most probable relaxation time membranes of the Nafion system doped with rare-earth metals: 1—Nafion, 2—Nafion + Tb, 3—Nafion + Eu.

The temperature dependence of the frequency (relaxation time) at which the maximum, corresponding to losses, *ν*_m_ (*τ*_m_), is observed (Figure 8) makes it possible to determine the number of relaxation processes and, if necessary, to determine the experimental activation energy *E_a_*, in other words, the energy barrier for dipole orientation.

### 3.2. Charge Transfer

**Figure 9:**
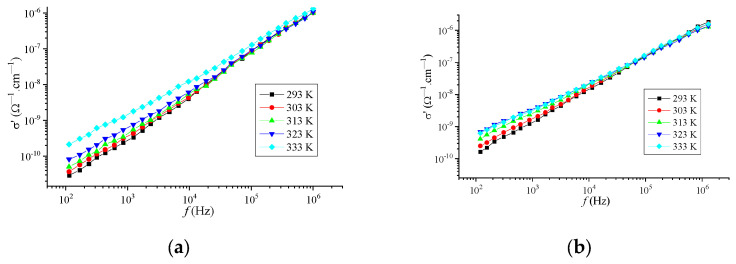
Frequency dependence of specific conductivity of Nafion + Tb (**a**), Nafion + Eu (**b**) systems at different temperatures.

**Figure 10:**
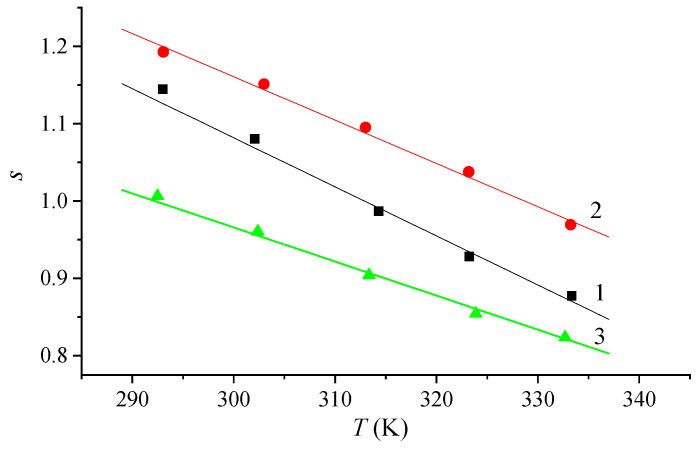
Temperature dependence of the exponent s for membranes of the Nafion system doped with rare-earth metals: 1—Nafion, 2—Nafion + Tb, 3—Nafion + Eu.

Figure 9 shows the frequency dependence of the conductivity at different temperatures for the modified Nafion samples. According to the figure, in the studied frequency range, the dispersion *σ′* obeys a power law:

(9) $$\sigma \prime \left(\omega \right)\sim \omega^{S},$$

which is typical for many disordered systems [38]. Here, ω is the angular frequency; *s* is the exponent, which decreases with increasing system temperature (Figure 10). The relative error in determining the value of the parameter s did not exceed 5% for all temperatures.

The power-law dependence of conductivity on frequency and the change in the value of the parameter *s* with increasing temperature make it possible to assume the existence of a hopping mechanism of conductivity, in which charge carriers (ions, electrons) make thermally activated jumps through the disordered structure of the systems [39].

For pure and modified Nafion membranes, the decrease in the value of *s* (Figure 10) is in good agreement with the concepts of the CBH model (correlated barrier hopping model) [40], according to which electrons jump between energy states, overcoming the potential barrier. In this case, the expression for conductivity on alternating current for a specific fixed temperature has the following form [41]:

(10) $$\sigma (\omega )=\frac{\pi^{3}N^{2}\varepsilon \varepsilon_{0}\omega R_{\omega }^{6}}{24}$$

where *N* is the density of states between which charge carriers jump, and *R_ω_* is the jump length.

The manifestation of the traditional mechanism of hopping conduction in these systems is connected with the fact that the displacement distance of the charge carrier is disproportionate to the time of application of the electric field at given temperatures [42,43]. As a result, the movement of the proton through the sample is limited. Therefore, not all protons approaching the surface will be able to overcome it.

**Figure 11:**
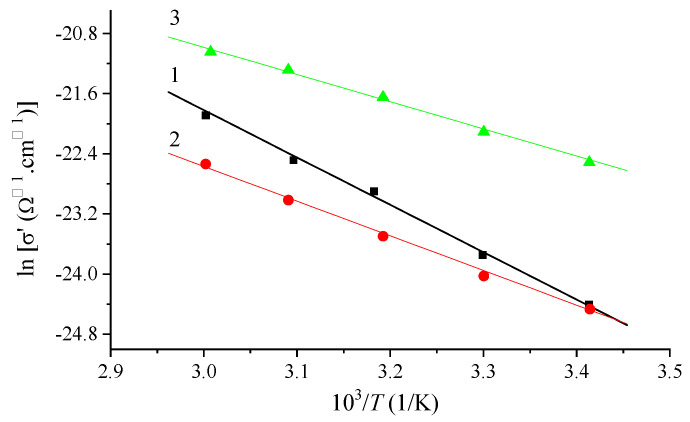
Temperature dependence of specific conductivity at a frequency *f* = 10^2^ Hz for three systems: 1—Nafion, 2—Nafion + Tb, 3—Nafion + Eu.

For all three systems, charge transfer is a thermally activated process (Figure 11). The existence of two temperature sections is observed; the first is a semiconductor section, and the second is a section of metallic conductivity. Table 2 shows the activation energy values obtained for the first section.

In the investigated range of frequencies and temperatures, it can be stated that the admixture of terbium reduces the conductivity of the Nafion layers, while the admixture of europium increases it. The decrease in conductivity by terbium can be explained by the adsorption of water in the volume of the polymer matrix and by a decrease in the number of protons due to their replacement by terbium ions. As for the increase in conductivity with europium, a stronger binding of europium ions to active sorption centers (sulfonic acid groups) in the membrane can be assumed. The strengthening of europium binding leads to a decrease in the distance between ions and to an increase in the charge density on the free surface, which leads to an increase in membrane conductivity.

## 4. Conclusions

The investigation results of the structural and dielectric properties of Nafion-type membranes are presented for the first time in this paper. Also, the relationship between their structure and relaxation properties was found.

The dispersion of the permittivity and the presence of maxima, corresponding to losses, indicate the existence of relaxation processes of various natures. A manifestation of the dipole relaxation polarization mechanisms associated with the relaxation of the polar groups in the system is observed. Undoped Nafion membranes are characterized by values of relaxation parameters that correspond to a symmetrical distribution of relaxers over relaxation times (beta is 1.0). The introduction of terbium and europium metal impurities into the polymer matrix leads to changes in the spectrum of relaxers, which manifests in the asymmetric nature of their distribution (for most temperatures, beta is different from 1.0).

For all systems under study, a power-law dependence of specific conductivity on frequency with the decrease in the exponent of power s with increasing temperature (s = 1.20…0.80) was found. These patterns indicate the existence of a classical hopping mechanism of charge transfer.

It is shown that for all three systems, charge transfer is a thermally activated process. The introduction of the impurities reduces the activation energy of conduction from 0.54 eV to 0.31 eV.

It can be assumed that the change in the dielectric permittivity and specific conductivity when Nafion membranes are doped with terbium and europium metals is due to a change in the nature of polarization processes because of the strong interaction of metal ions with the polymer matrix.

The results of IR measurements and adsorption measurements indicate the similarity between the studied membranes and Nafion. The increase in the conductivity of systems with Eu (3+) (in comparison with Tb (3+)) can be associated with a stronger binding of europium ions and the increase in charge density.

## Figures and Tables

**Table 1:** Parameters of the Havriliak–Negami function for samples of three systems.

System	Temp., K	−lg τ_max_ (s)	D_Eps	Alpha	Beta
Nafion	293	5.98005	6.6150 × 10^0^	5.7810 × 10^−1^	1.0000 × 10^0^
	303	6.07242	7.3550 × 10^0^	5.6600 × 10^−1^	1.0000 × 10^0^
	313	6.03194	8.0440 × 10^0^	5.4930 × 10^−1^	9.8960 × 10^−1^
	323	6.21226	9.5670 × 10^0^	5.8080 × 10^−1^	1.0000 × 10^0^
	333	6.31229	1.0250 × 10^0^	7.1850 × 10^−1^	1.0000 × 10^0^
Nafion + Tb	293	6.38132	9.9430 × 10^0^	4.7640 × 10^−1^	9.5980 × 10^−1^
	303	6.40738	9.3940 × 10^0^	4.9860 × 10^−1^	9.8310 × 10^−1^
	313	6.4618	8.8800 × 10^0^	5.1050 × 10^−1^	1.0000 × 10^0^
	323	6.41285	9.7360 × 10^0^	5.1860 × 10^−1^	9.4930 × 10^−1^
	333	6.2609	1.2990 × 10^1^	5.0100 × 10^−1^	1.0000 × 10^0^
Nafion + Eu	293	6.91045	2.9270 × 10^1^	2.8880 × 10^−1^	9.9590 × 10^−1^
	303	6.75771	2.4060 × 10^1^	3.5570 × 10^−1^	1.0000 × 10^0^
	313	6.80493	2.4640 × 10^1^	3.3420 × 10^−1^	1.0000 × 10^0^
	323	6.78146	2.4620 × 10^1^	3.6500 × 10^−1^	7.3340 × 10^−1^
	333	6.82246	2.6840 × 10^1^	3.5770 × 10^−1^	8.5010 × 10^−1^

**Table 2:** The values of the activation energy of the conductivity in the semiconductor section.

System	Nafion	Nafion + Tb	Nafion + Eu
Activation energy (eV)	0.54	0.39	0.31
Accuracy (±)	0.02	0.01	0.01

## Data Availability

The data presented in this study are available in this article.
